# United States Public Health Service

**Published:** 1916-09

**Authors:** 


### United States Public Health Service.

WASHINGTON, August 7, 1916.

Congress has recently made an appropriation for thirty-three additional assistant surgeons in the United States Public Health Service. These officers are commissioned by the President and confirmed by the Senate. The tenure of office is permanent, and successful candidates will immediately receive their commissions.

After four years' service, assistant surgeons are entitled to examinations for promotion to the grade of passed assistant surgeon.

Examinations will be held every month or so in various cities. For further information, write the Surgeon General, United States Public Health Service, Washington, D. C.

